# Carotenoid metabolite and transcriptome dynamics underlying flower color in marigold (*Tagetes erecta* L.)

**DOI:** 10.1038/s41598-020-73859-7

**Published:** 2020-10-08

**Authors:** Huali Zhang, Shiya Zhang, Hua Zhang, Xi Chen, Fang Liang, Helan Qin, Yue Zhang, Richen Cong, Haibo Xin, Zhao Zhang

**Affiliations:** 1 Beijing Key Laboratory of Greening Plants Breeding, Beijing Institute of Landscape Architecture, Beijing, 100102 China; 2 Beijing Key Laboratory of Development and Quality Control of Ornamental Crops, Department of Ornamental Horticulture, China Agricultural University, Yuanmingyuan Xilu 2, Beijing, 100193 China; 3 Jiangsu Vocational College of Agriculture and Forestry, Nanjing, China

**Keywords:** Plant sciences, Secondary metabolism

## Abstract

Marigold (*Tagetes erecta* L.) is an important ornamental plant with a wide variety of flower colors. Despite its economic value, few biochemical and molecular studies have explored the generation of flower color in this species. To study the mechanism underlying marigold petal color, we performed a metabolite analysis and de novo cDNA sequencing on the inbred line ‘V-01’ and its petal color mutant ‘V-01M’ at four flower developmental stages. A total of 49,217 unigenes were identified from 24 cDNA libraries. Based on our metabolites and transcriptomic analyses, we present an overview of carotenoid biosynthesis, degradation, and accumulation in marigold flowers. The carotenoid content of the yellow mutant ‘V-01M’ was higher than that of the orange inbred line ‘V-01’, and the abundances of the yellow compounds lutein, neoxanthin, violaxanthin, zeaxanthin, and antheraxanthin were significantly higher in the mutant. During flower development, the carotenoid biosynthesis genes were upregulated in both ‘V-01’ and ‘V-01M’, with no significant differences between the two lines. By contrast, the carotenoid degradation genes were dramatically downregulated in the yellow mutant ‘V-01M’. We therefore speculate that the carotenoid degradation genes are the key factors regulating the carotenoid content of marigold flowers. Our research provides a large amount of transcriptomic data and insights into the marigold color metabolome.

## Introduction

Carotenoids play an important role in photosynthesis, and their degradation produces a series of plant volatiles, as well as strigolactones and abscisic acid phytohormones^1,2^. Moreover, carotenoids are widely used in the food and pharmaceutical industry; for example, lutein and similarly structured carotenoids can protect retinal cells in the eye against oxidative stress, and a number of studies have suggested that the supplementation of lutein can maintain eye health and lower the risk of various chronic eye diseases^3^.

Marigold (*Tagetes erecta* L.) is native to Mexico and South America^4^. In addition to its use as a potted plant, a landscaping plant, and a cut flower, marigold is an important source of lutein. The consumption of lutein-rich foods can effectively reduce the chance of developing macular degeneration, cataracts, and atherosclerosis, as well as the development of certain cancers^5,6^; therefore, the international demand for lutein is constantly increasing. Lutein is the main pigment in marigold petals, accounting for up to 90% of the total carotenoids in these flowers^7^. Marigold is one of the main raw materials from which lutein is extracted, making it a promising cash crop.

In plants, the precursor of carotenoid biosynthesis is derived from the 2-C-methyl-d-erythritol-4-phosphate (MEP) pathway, which takes place in the plastids. The biosynthesis of the first carotenoid compound precursor, phytoene, requires four enzymes: 1-deoxylulose-5-phosphate synthase (DXS), 1-deoxylulose-5-phosphate reductionomerase (DXR), geranylgeranyl pyrophosphate synthase (GGPPS), and phytoene synthase (PSY). Another four enzymes participate in the catalytic reaction to transform colorless phytoene into red lycopene, namely phytoene desaturase (PDS), 15-cis-ζ-carotene isomerase (Z-ISO), ζ-carotene desaturase (ZDS), and carotenoid isomerase (CRTISO). After the formation of lycopene, the metabolic pathway divides into two branches. One of these branches results in the catalysis of lycopene by β-cyclase (LCY-B) and β-hydroxylase (HYD-B) to produce zeaxanthin, while the other branch involves LCY-B, HYD-B, ε-cyclase (LCY-E), and ε-hydroxylase (HYD-E), which function to produce lutein. In marigold flowers, *LCY-E* is expressed in the petals, and its expression is positively associated with lutein accumulation^8^.

The accumulation of lutein in plants is also determined by its degradation. The degradation of lutein and other carotenoids involves the carotenoid cleavage dioxygenase (CCD)^9^ and 9-*cis* epoxy carotenoid cleavage dioxygenase (NCED) enzymes^10^. CCD performs a major role in the degradation of a series of xanthophylls, such as lutein, zeaxanthin, violaxanthin, neoxanthin, and antheraxanthin, whereas NCED specifically catalyzes the degradation of zeaxanthin.

In general, genes encoding carotenoid biosynthesis and degradation enzymes are expressed in specific patterns in the various organs and at different developmental stages. Their expression patterns are finely regulated by various transcription factors, but only a few studies have explored these regulatory pathways in detail^11^. Ralf et al.^12^ found that an *Arabidopsis thaliana* APETALA2 (AP2)/ethylene-responsive element-binding protein transcription factor (AP2/ERF), RAP2.2, binds to the *PSY* and *PDS* promoters. The *rap2.2* knockdown mutant displayed a decreased level of *PSY* and *PDS* expression, in addition to a 30% reduction in its carotenoid content. A phytochrome-interacting factor (PIF) was also shown to bind to the *PSY* promoter and inhibit its expression^13^. Similarly, a MADS box transcription factor, RIN (ripening inhibitor), was found to interact with the *PSY* promoter and participate in carotenoid accumulation in tomato (*Solanum lycopersicum*) fruit^14^. These transcription factors are known to regulate carotenoid accumulation in general; however, no transcription factors have been reported to regulate lutein biosynthesis or degradation specifically.

The lutein contents of different marigold varieties can vary more than 100 fold, resulting in differing petal colors, which can be white, cream, yellow, and orange-red. We previously developed the marigold inbred line ‘V-01’, which has orange petals, and recently identified a natural mutant derived from this population, ‘V-01M’, which displayed identical developmental and botanical characteristics to ‘V-01’, except that it produced yellow petals. Here, we used metabolome and transcriptome sequencing techniques combined with bioinformatics to analyze these two marigold genotypes and identify the genetic mechanisms underpinning their different flower colors. This work improves our understanding of the transcriptional mechanisms by which carotenoid accumulation and degradation are regulated.

## Results

### Carotene and xanthophyll accumulation in the petals of ‘V-01’ and its natural mutant ‘V-01M’

**Figure 1:**
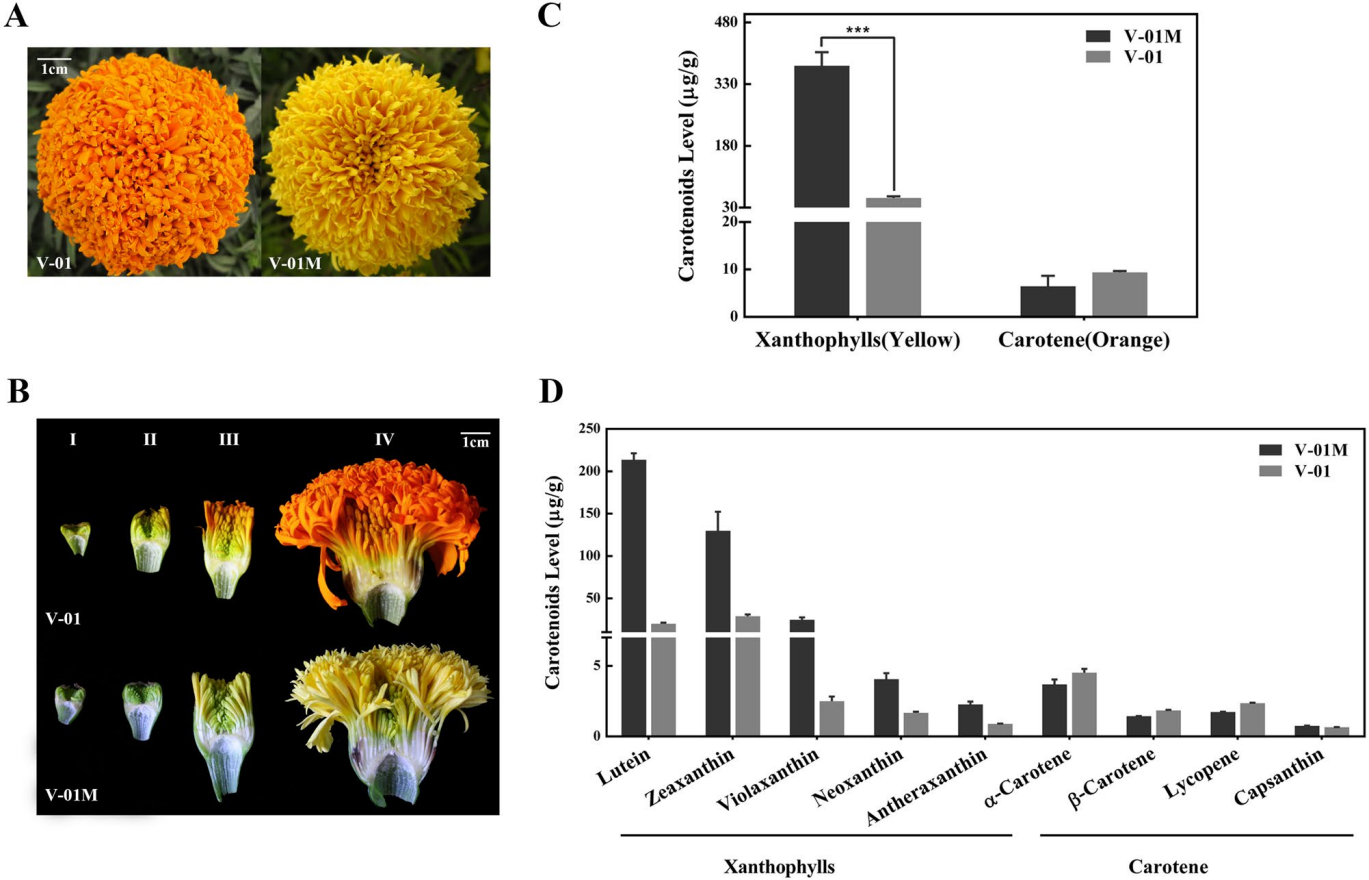
Carotenoid accumulation in ‘V-01’ and ‘V-01M’ marigold petals. (**A**) The flowers of the ‘V-01’ line are orange, while its natural mutant ‘V-01M’ is yellow. (**B**) The four stages of floral development in marigold ‘V-01’ and ‘V-01M’, shown using longitudinal cross-sections of the flowers. (**C**) The accumulation of carotenoids in the flowers of ‘V-01’ and ‘V-01M’ at developmental Stage IV. The error bars represent the standard error of three biological replicates. Asterisks indicate significant differences according to a Student’s *t*-test (*P < 0.001). (**D**) The accumulation of xanthophylls and carotenes in marigold ‘V-01’ and ‘V-01M’ at developmental Stage IV. The error bars represent the standard error of three biological replicates.

The marigold inbred line ‘V-01’ (orange flowers) and its natural mutant ‘V-01M’ (yellow flowers) had very similar botanical characteristics, except for their petal color (Fig. 1A). Marigold flower development can be divided into four stages (Fig. 1B); in the first stage, the ligulate flowers are tightly packed and green (Stage I), after which the outermost ligulate flowers begin to expand (Stage II). Next, the ligulate flowers elongate, with pigmentation starting to appear from the outermost layer (Stage III), and finally, all of the ligulate flowers expand and spread evenly to form the marigold inflorescence (Stage IV). We found that the orange variety ‘V-01’ and its natural mutant ‘V-01M’ had visible color differences starting from Stage III, resulting in completely different flower colors at Stage IV.

To analyze the differences in color at the biochemical level, we assessed the accumulation of carotenoids in the Stage-IV ligulate flowers of ‘V-01’ and ‘V-01M’ using HPLC and mass spectrometry. A total of nine carotenoids were detected, which could be divided into two subgroups: carotenes (orange pigments) and xanthophylls (yellow pigments). The carotenes included α-carotene, β-carotene, lycopene, and capsanthin, while the xanthophylls included lutein, violaxanthin, zeaxanthin, neoxanthin, and antheraxanthin. The xanthophylls was significantly more abundant in the yellow mutant ‘V-01M’ than in the orange line ‘V-01’ (Fig. 1C,D). In ‘V-01M’, the contents of all five xanthophylls were significantly higher than in ‘V-01’, especially for lutein and zeaxanthin (Fig. 1D). In contrast, no significant difference of carotenes were detected in ‘V-01’ and ‘V-01M’ (Fig. 1C). These results showed that the higher accumulation of yellow pigments (xanthophylls) in the ‘V-01M’ mutant likely resulted in its yellow petal color. This accumulation of xanthophylls could be caused by the promotion of the biosynthesis pathway or the repression of the degradation pathway.

### Illumina sequencing, de novo assembly, and functional annotation

**Figure 2:**
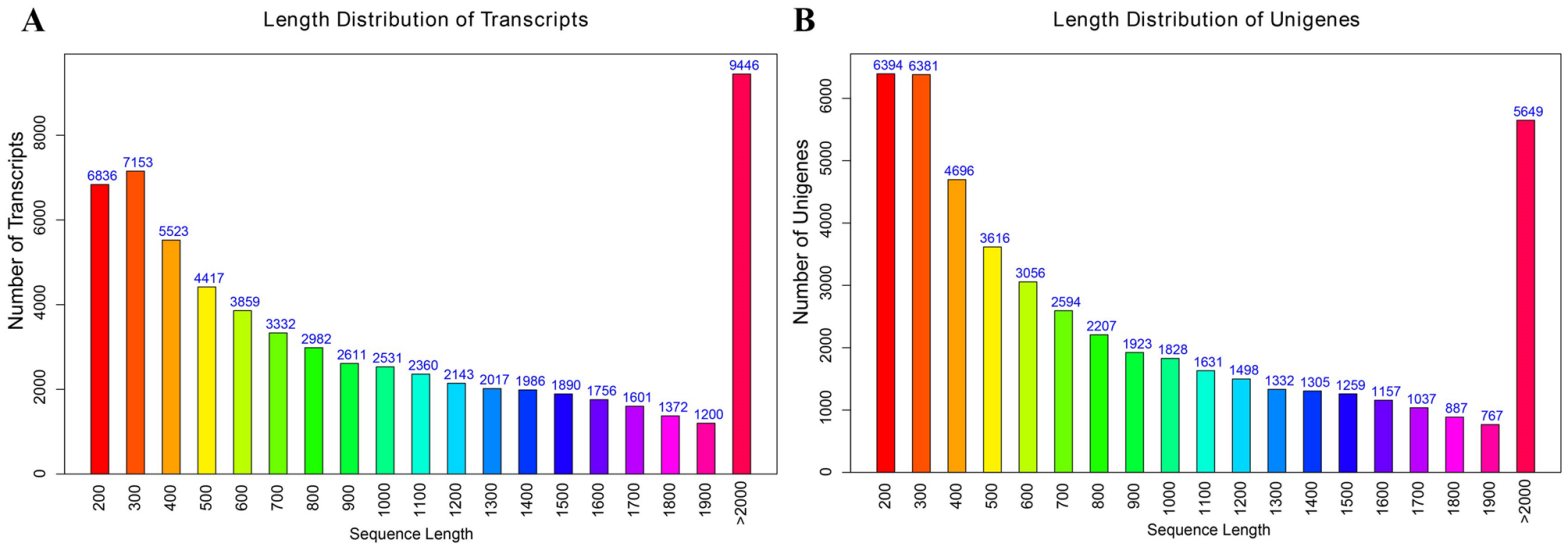
Length frequency distribution of assembly transcripts and unigenes. (**A**) Length distribution of transcripts. (**B**) Length distribution of unigenes.

To elucidate the mechanism of flower color biosynthesis and carotenoid metabolism in marigold, we conducted the de novo sequencing of the orange and yellow varieties. A total of 24 RNA libraries were constructed from the flowers of the two lines (‘V-01’ and ‘V-01M’) at the four developmental stages, with three biological replicates for each stage (Supplementary Table S1). We obtained 132.954 Gb of clean data, which were used to assemble a de novo transcriptome using Trinity. The assembly results led to the identification of 65,015 transcripts with an average length of 1130 bp, a GC content of 39.75%, and a N50 score of 1635 bp. These 65,015 transcripts belonged to 49,217 unigenes, which had an average length of 1015 bp, a GC content of 40.1%, and a N50 score of 1501 bp (Table 1). The size distribution of the transcripts and unigenes are given in Fig. 2, with 43.53% and 37.28% of all transcripts and unigenes showing lengths greater than 1 kb, respectively.

**Table 1:** Length distribution of assembly transcripts and unigenes.

	All	≥ 500 bp	≥ 1000 bp	N50	GC (%)	Total length	Max. length	Min. length	Average length
Transcript	65,015	45,503	28,302	1635	39.75	73,506,795	13,450	224	1130
Unigene	49,217	31,746	18,350	1501	40.1	49,955,300	13,450	224	1015

**Figure 3:**
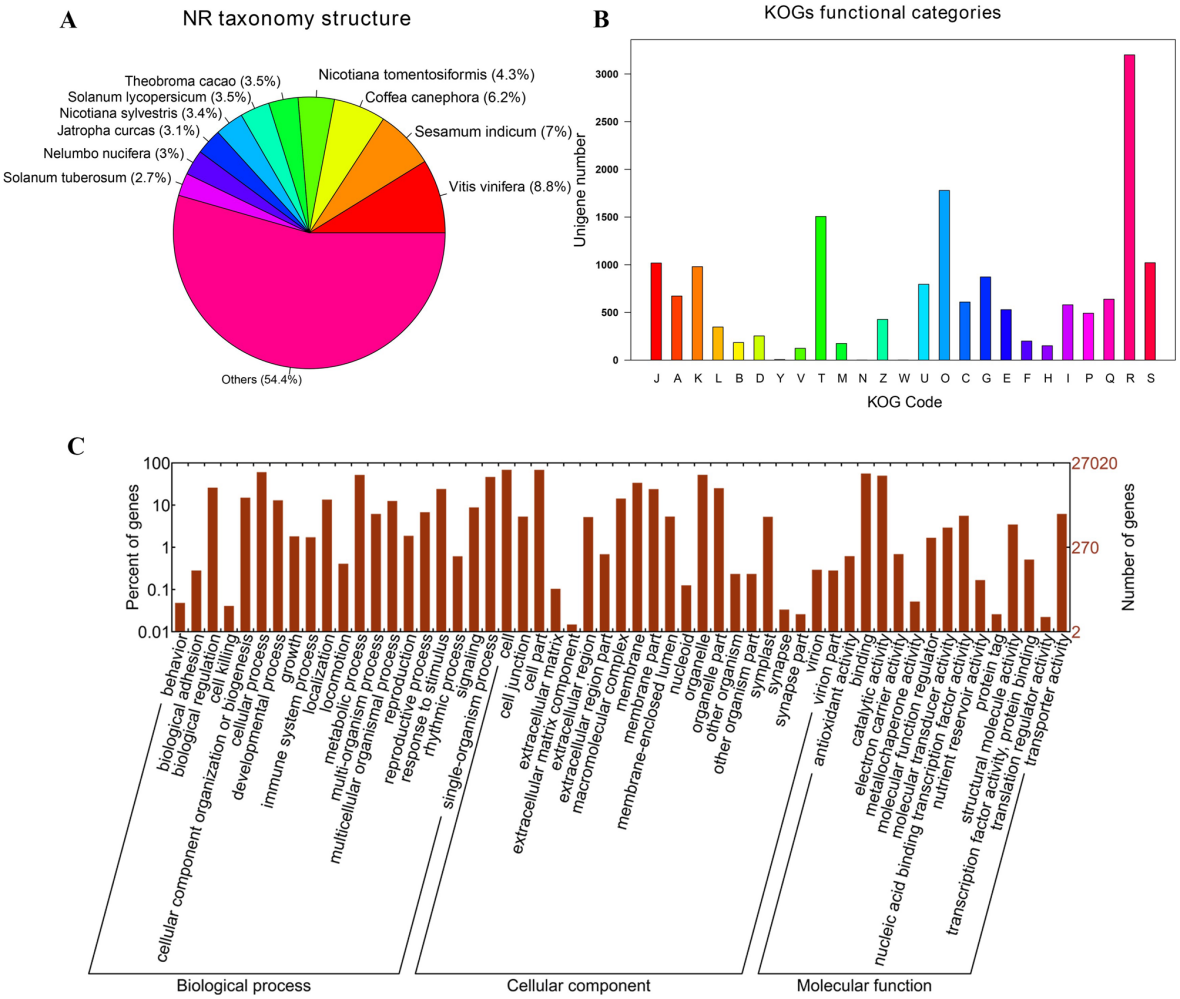
Characteristic analysis of annotated unigenes. (**A**) Species distribution of the top BLAST hits for each unique sequence. (**B**) Functional classification of marigold unigenes in KOG categories. The categories: J, Translation, ribosomal structure and biogenesis; A, RNA processing and modification; K, Transcription; L, Replication, recombination and repair; B, Chromatin structure and dynamics; D, Cell cycle control, cell division, chromosome partitioning; Y, Nuclear structure; V, Defense mechanisms; T, Signal transduction mechanisms; M, Cell wall/membrane/envelope biogenesis; N, Cell motility; Z, Cytoskeleton; W, Extracellular structures; U, Intracellular trafficking, secretion, and vesicular transport; O, Posttranslational modification, protein turnover, chaperones; C, Energy production and conversion; G, Carbohydrate transport and metabolism; E, Amino acid transport and metabolism; F, Nucleotide transport and metabolism; H, Coenzyme transport and metabolism; I, Lipid transport and metabolism; P, Inorganic ion transport and metabolism; Q, Secondary metabolites biosynthesis, transport and catabolism; R, General function prediction only; S, Function unknown. (**C**) Functional classification of marigold unigenes enriched in GO terms.

Gene function was annotated based on the homology of the unigenes to sequences listed in the following databases: Swiss_Prot, TrEMBL, NR, Pfam, KOG, GO, and KEGG. Putative homologs were identified for 33,810 of the unigenes (68.70%) in the NR database, while 33,646 (68.40%), 27,502 (55.90%) 28,629 (58.20%), 22,558 (45.8%), 27,020 (54.90%), and 9790 (19.90%) unigenes showed significant similarity to sequences in the TrEMBL, Pfam, KOG, Swiss_Prot, GO, and KEGG databases, respectively (Table 2). Among the 33,810 unigenes with a match in the NR database, 8.8% were most similar to sequences from grape (*Vitis vinifera*), followed by sesame (*Sesamum indicum*; 7%), robusta coffee (*Coffea canephora*; 6.2%), and wild tobacco (*Nicotiana tomentosiformis*; 4.3%) (Fig. 3A). The predicted function and gene classification of the marigold unigenes were identified using the KOG and GO databases. A total of 1779 unigenes were annotated as ‘signal transduction mechanisms’ based on the KOG database, and the most common category was ‘general function prediction only’ (3200 unigenes) (Fig. 3B). Furthermore, the unigenes were annotated with GO terms, with the most common biological process categories determined to be ‘metabolic process’ and ‘cellular process’ (Fig. 3C).

**Table 2:** Number of unigenes annotated using homology to sequences in public databases^a^.

Unigenes	Swiss_Prot	TrEMBL	NR	Pfam	KOG	GO	KO
49,217	22,558	33,646	33,810	27,502	28,629	27,020	9790
100%	45.80%	68.40%	68.70%	55.90%	58.20%	54.90%	19.90%

^a^The public databases are: Swiss_Prot; Translated EMBL, TrEMBL; Non-Redundant Protein Sequence Database, NR; Protein families and domain database, Pfam; euKaryotic Orthologous Groups database, KOG; Gene Ontology, GO; KEGG orthology database, KO.

### Expression dynamics of the DEGs during flower development

**Figure 4:**
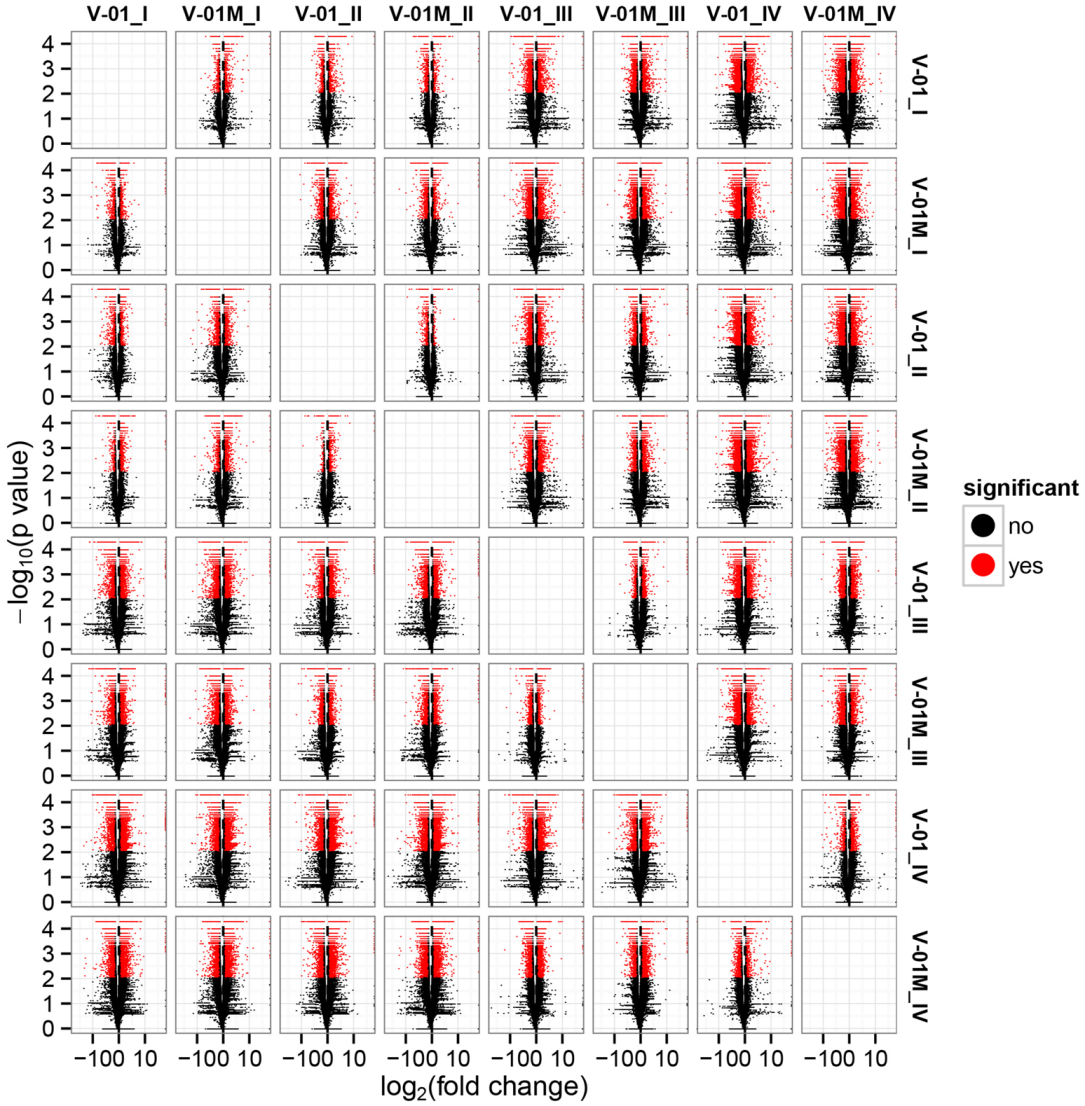
Volcano map of differentially expressed genes in a comparison of 24 libraries, which were constructed from the flowers of the two lines (‘V-01’ and ‘V-01M’) at the four developmental stages (e.g. V-01_I indicates the flower development stage I of V-01 and so on). The abscissa represents the level of fold change of the differentially expressed genes. The ordinates represents the significance level of the gene expression changes. Significantly differentially expressed genes are shown as red dots, while those with no significant difference are shown as black dots.

Cufflinks software was used to identify DEGs between the four developmental stages in both of the marigold genotypes. The FPKM values were used to estimate the gene expression levels, and volcano plots were constructed to describe the distribution of all DEGs identified in the library comparisons (Fig. 4). These results indicated that, in both ‘V-01’ and ‘V-01M’, the most dramatic change in the expression of the genes occurred between developmental Stages I to III, as well as the comparison between Stages I and IV. This suggested that a large number of genes are significantly differentially expressed throughout flower development. Furthermore, the fold changes in the expression of the DEGs between Stages II and III were greater than those of the DEGs from the comparison of Stages I and II in both ‘V-01’ and ‘V-01M’, suggesting that a more dramatic change in gene expression occurred between Stages II and III than between Stages I and II.

**Figure 5:**
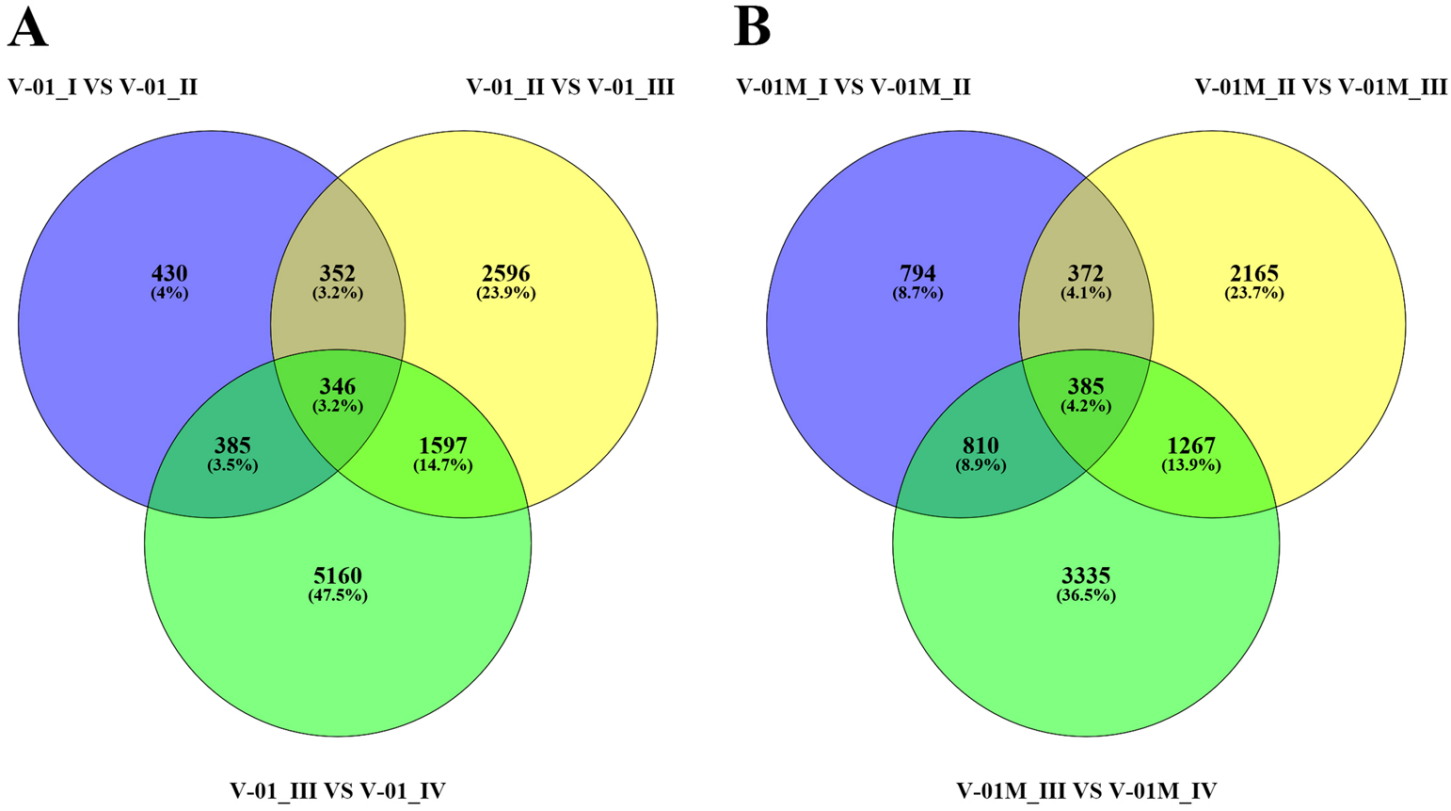
Venn diagram of the number of differentially expressed genes in the four stages of marigold (**A**) ‘V-01’ and (**B**) ‘V-01M’ flower development. The genes in overlapping sets show a differential expression in two or three comparison pairs.

Similarly, significantly fewer DEGs (1513) were identified in V-01_I_VS_V-01_II (the comparison between Stages I and II in ‘V-01’) than in V-01_II_VS_V-01_III (4891) and V-01_III_VS_V-01_IV (7488), and comparisons of these stages in the mutant plant ‘V-01M’ also followed a similar DEG pattern (Fig. 5; Supplemental Table S2). This suggested that Stages II and III are the key phases of flower development with the most dramatic changes in gene expression. This is consistent with the observed accumulation of carotenoids and the color changes in the marigold flowers beginning in Stage III (Fig. 1B). The coloring of the ligulate marigold flowers began at Stage III, and the ‘V-01’ (orange) and ‘V-01M’ (yellow) began to visibly differentiate during this stage.

The most dramatic changes in gene expression, both in fold change (Fig. 4) and the number of DEGs (Fig. 5), occurred between the flower development Stages II and III. We therefore performed a detailed comparative analysis of the DEGs in Stages II and III (V-01_II_VS_V-01_III and V-01M_II_VS_V-01M_III), during which the ligulate flowers transitioned into the critical period of color formation. Finally, 4891 DEGs (2369 upregulated and 2522 downregulated) were identified in the V-01_II_VS_V-01_III comparison and 4189 (2453 upregulated and 1736 downregulated) were identified in the V-01M_II_VS_V-01M_III comparison (Fig. 5; Supplemental Table S2). The similar number of DEGs between these two comparisons was likely a reflection of their similar genetic backgrounds.

**Figure 6:**
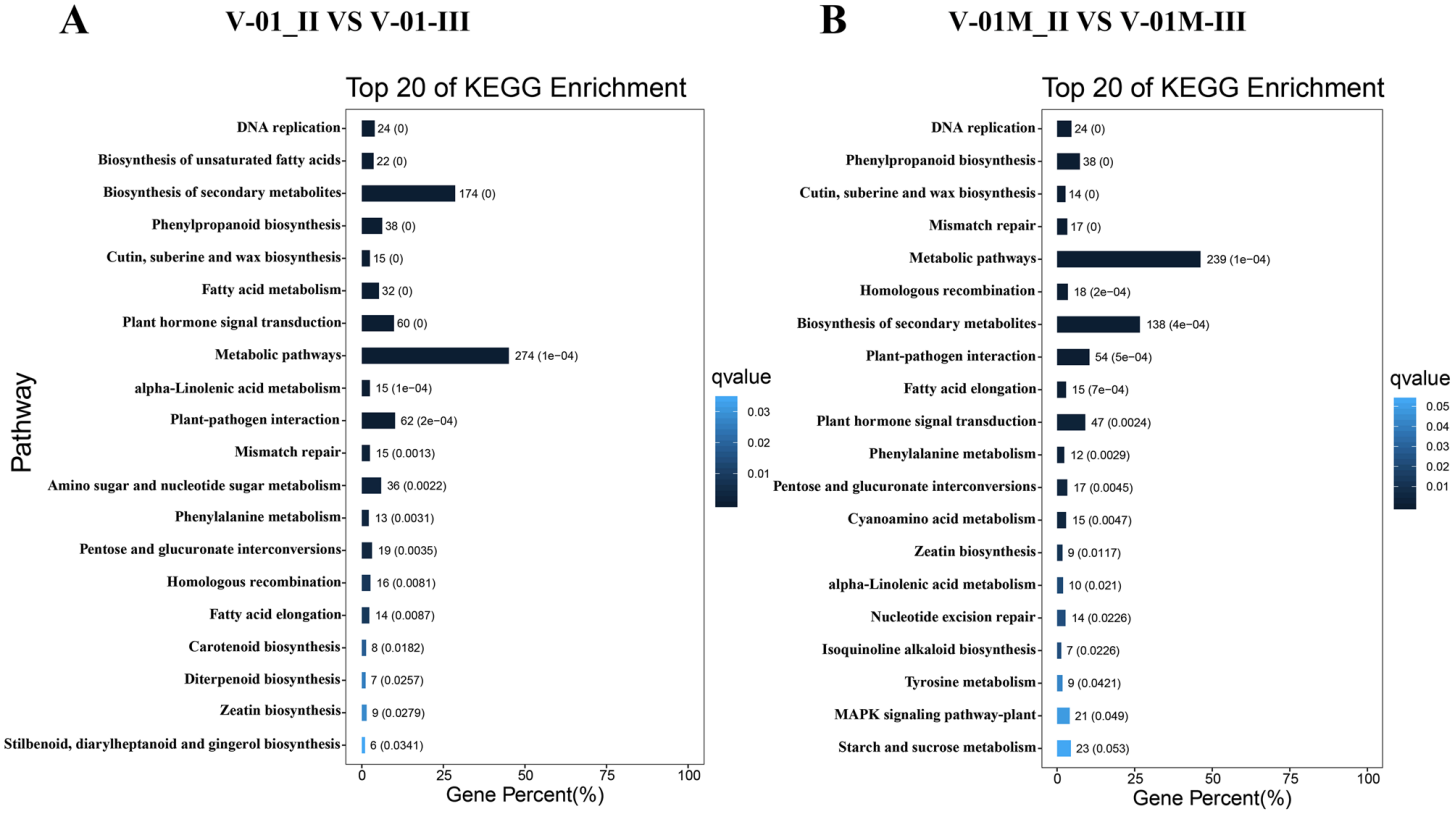
KEGG enrichment of differentially expressed genes in a comparison between Stages II and III of marigold flower development in ‘V-01’ and ‘V-01M’.

We further used a KEGG analysis for the functional classification and pathway assignment of the DEGs between Stages II and III in both ‘V-01’ and ‘V-01M’. For V-01_II_VS_V-01_III, a total of 904 upregulated DEGs and 609 downregulated DEGs were grouped into the KEGG pathways. Similarly, 778 upregulated DEGs and 519 downregulated DEGs from the V-01M_II_VS_V-01M_III comparison were grouped into the KEGG pathways. The most significantly enriched pathways associated with the DEGs were “metabolic pathways” and “biosynthesis of secondary metabolites” in both ‘V-01’ and ‘V-01M’ (Fig. 6), suggesting a potentially important role for secondary metabolites in floral development.

### Identification of genes involved in carotenoid biosynthesis and degradation

Carotenoids are major pigments in marigold flowers, and the differing carotene (orange pigments) and xanthophyll (yellow pigments) contents in different genotypes largely contribute to the diversity of their flower colors. According to the KOG classification, about 2.2% of transcripts in the marigold flowers were assigned to the secondary metabolite biosynthesis category (Fig. 3B). Many of these encoded enzymes known to catalyze the biosynthesis of various carotenoids, including α-carotene, β-carotene, lycopene, capsanthin, lutein, violaxanthin, zeaxanthin, neoxanthin, and antheraxanthin.

**Figure 7:**
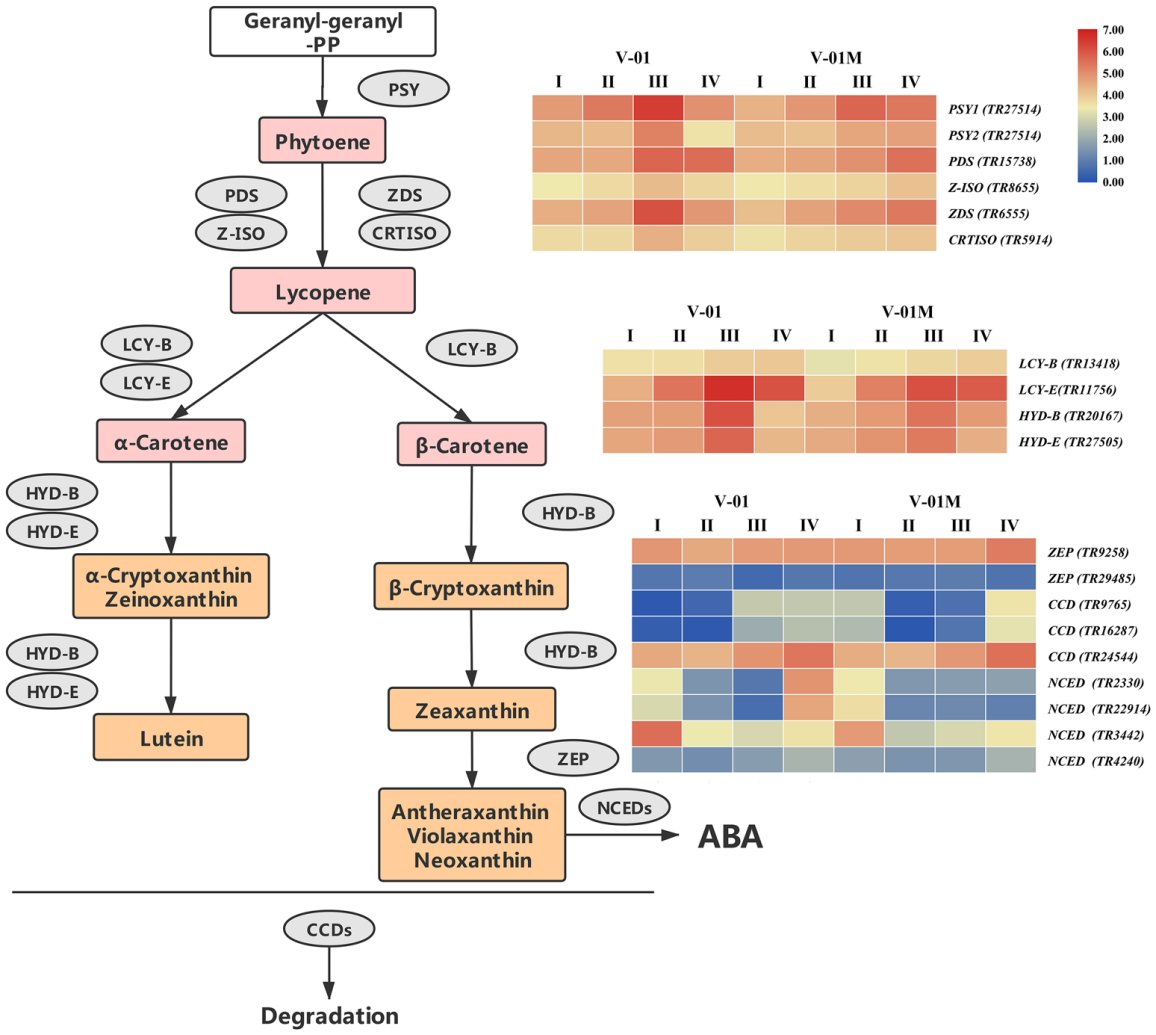
Expression patterns of genes encoding enzymes putatively involved in the biosynthesis and degradation of carotenoids in the marigold flowers. Gene expression levels in the four flower developmental stages in both marigold ‘V-01’ and ‘V-01M’ are represented by heat maps. *PSY* phytoene synthase, *PDS* phytoene desaturase, *Z-ISO* 15-*cis*-zeta-carotene isomerase, *ZDS* zeta-carotene desaturase, *CRTISO* prolycopene isomerase, *LCY-B* lycopene beta cyclase, *LCY-E* lycopene epsilon cyclase, *HYD-B* beta-ring hydroxylase, *HYD-E* carotene epsilon-monooxygenase, *ZEP* zeaxanthin epoxidase, *CCD* carotenoid cleavage dioxygenase, *NCED* 9-*cis*-epoxycarotenoid dioxygenase.

To elucidate the genetic regulatory mechanism of marigold pigment accumulation, the genes involved in the carotenoid biosynthesis pathway were identified according to the annotations of the transcriptome data. The first step in carotenoid biosynthesis is the conversion of colorless phytoene to red lycopene. The enzymes involved in this step were identified in the marigold transcriptomes, including *PDS* (TR15738), *Z-ISO* (TR8655), *ZDS* (TR6555), and *CRTISO* (TR5914). Lycopene is then catalyzed into carotene by the cyclases LCY-B and LCY-E, which were also identified in the marigold transcriptomes (*LCY-B* (TR13418) and *LCY-E* (TR11756)). Carotene can be further catalyzed to produce a number of xanthophylls, including lutein, zeaxanthin, violaxanthin, and neoxanthin, which involves the genes *HYD-B* (TR20167) and *HYD-E* (TR27505). Finally, in addition to their biosynthesis pathway, three *CCD* genes (TR9765, TR16287, and TR24544) and four *NCED* genes (TR2330, TR3442, TR4240, and TR22914), all of which catalyze the degradation of the carotenoids, were identified in the transcriptome data (Table 3). Among the three identified *CCD* genes, TR9765 is phylogenetically close to Arabidopsis *AtCCD1*, whereas both TR16287 and TR24544 are close to *AtCCD7*. The enzymes encoded by these proteins are likely involved in the biosynthesis and degradation of carotenoids. The change in their expression may therefore be vital for the final color of the marigold flowers (Fig. 7).

**Table 3:** List of unigenes involved in the carotenoid metabolic pathway.

Unigene	Gene	Definition	Length (bp)
TR15738	*PDS*	Phytoene desaturase	1993
TR8655	*Z-ISO*	15-*Cis*-zeta-carotene isomerase	1272
TR6555	*ZDS*	Zeta-carotene desaturase	2065
TR5914	*CRTISO*	Prolycopene isomerase	2138
TR13418	*LCY-B*	Lycopene beta cyclase	1956
TR11756	*LCY-E*	Lycopene epsilon cyclase	3295
TR20167	*HYD-B*	Beta-ring hydroxylase	2095
TR27505	*HYD-E*	Carotene epsilon-monooxygenase	1931
TR9765	*CCD*	Carotenoid 9,10(9′,10′)-cleavage dioxygenase	756
TR16287	*CCD*	Carotenoid 9,10(9′,10′)-cleavage dioxygenase	841
TR24544	*CCD*	Carotenoid 9,10(9′,10′)-cleavage dioxygenase	1886
TR2330	*NCED*	9-*Cis*-epoxycarotenoid dioxygenase	884
TR3442	*NCED*	9-*Cis*-epoxycarotenoid dioxygenase	2200
TR4240	*NCED*	9-*Cis*-epoxycarotenoid dioxygenase	1860
TR22914	*NCED*	9-*Cis*-epoxycarotenoid dioxygenase	1324

### Transcriptome dynamics of genes involved in carotenoid biosynthesis

During the four stages of flower development, most of the genes involved in carotenoid biosynthesis and degradation were differentially regulated. Four genes encoding enzymes that catalyze the conversion of phytoene into lycopene, *PDS* (TR15738), *Z-ISO* (TR8655), *ZDS* (TR6555), and *CRTISO* (TR5914), were significantly upregulated in Stages III and IV (Fig. 7). Similarly, *LCY-B* (TR13418), *LCY-E* (TR11756), *HYD-B* (TR20167), and *HYD-E* (TR27505) were upregulated in Stages III and IV. The upregulation of the carotenoid biosynthesis genes probably reflects the pigmentation of the flowers at developmental Stages III and IV, during petal expansion. Both ‘V-01’ and ‘V-01M’ have a similar expression pattern of these genes.

### Carotenoid degradation genes are differentially expressed between ‘V-01’ and ‘V-01M’

To determine the causal genes responsible for the different accumulation patterns of carotenoids in ‘V-01’ and ‘V-01M’, we compared their expression patterns of the carotenoid biosynthesis genes. The genes catalyzing phytoene to lycopene, including *PDS* (TR15738), *Z-ISO* (TR8655), *ZDS* (TR6555), and *CRTISO* (TR5914), are more highly expressed in ‘V-01’ than ‘V-01M’ in developmental Stages III and IV (Fig. 7).

The ‘V-01M’ flowers accumulated significantly more xanthophylls than ‘V-01’ (more than a ten-fold difference); therefore, we also investigated their expression of *HYD-B* (TR20167) and *HYD-E* (TR27505), which are involved in the biosynthesis of a series of xanthophylls. Surprisingly, no significant difference was observed in the expression of either *HYD-B* or *HYD-E* between ‘V-01’ and ‘V-01M’ in any of the four developmental stages, suggesting that this is not the reason for the color differences observed in these lines.

We next examined the expression of the genes responsible for xanthophyll degradation, the most important of which encode the CCD enzymes. Among the three *CCD* genes identified in our transcriptome data, TR9765 was noticeably downregulated (8.96-fold decrease) in ‘V-01M’ compared with ‘V-01’ at developmental Stage III. Similarly, another *CCD* gene, TR16287, was expressed to a level 4.30 times lower in ‘V-01M’ than in ‘V-01’ at developmental Stage III (Fig. 7). In contrast, these two genes are less down-regulated in ‘V-01M’ compared with ‘V-01’ in stage IV. This may be involved in feedback regulation of degradation genes reflex by the high accumulation of carotenoid in ‘V-01M’.

In addition to the CCDs, the degradation of zeaxanthin also involves the NCEDs. At developmental Stage IV, two of the four *NCED* genes identified in the marigold transcriptome were expressed at a dramatically lower level in ‘V-01M’ than in ‘V-01’; 22.62-fold and 12.35-fold decreases were observed in the expression of *TR22914* and *TR2330*, respectively, in the mutant flowers (Fig. 7).

The CCDs and NCEDs are the enzymes responsible for the degradation of xanthophylls; therefore, the low expression of *CCD*s and *NCED*s in the ‘V-01M’ mutant likely resulted in its accumulation of xanthophylls and consequently the yellow color of its flowers.

## Discussion

Carotenoids play an important role in photosynthesis, and their degradation produces a series of plant volatiles, as well as strigolactones and abscisic acid phytohormones^1,2^. Moreover, carotenoids are widely used in the food and pharmaceutical industry; for example, lutein and similarly structured carotenoids can protect retinal cells in the eye against oxidative stress, and a number of studies have suggested that the supplementation of lutein can maintain eye health and lower the risk of various chronic eye diseases^3^. Lutein is the major pigment in marigold petals, making this plant one of the most important sources of this xanthophyll in the pharmaceutical industry. In some cultivars, lutein can account for approximately 90% of the total carotenoids in the marigold petals^7^; however, the lutein contents of the different varieties of marigold can vary substantially, with more than 100-fold differences detected between some lines^8^. Many studies have therefore examined the genetic regulation of carotenoid accumulation in marigolds, which are considered a model plant for analysis of this pathway.

We further elucidated the marigold carotenoid metabolic pathway by comparing an orange inbred line ‘V-01’ and its yellow mutant ‘V-01M’. A number of carotenoid substances were detected in both marigold lines, with the xanthophylls such as lutein, zeaxanthin, antheraxanthin, neoxanthin, and violaxanthin being significantly more abundant in the yellow mutant ‘V-01M’ than in ‘V-01’. These yellow substances accounted for 95.98% of the total carotenoid content in ‘V-01M’ but only 40.44% of the total carotenoid content in ‘V-01’, suggesting that changes in the relative abundance of the carotenoids directly affect the color of marigold flowers.

To explore the genetic pathways involved in carotenoid accumulation in ‘V-01’ and ‘V-01M’, we performed transcriptome sequencing and a differential expression analysis. Few previous studies have examined the marigold transcriptome, and fewer still have explored the transcriptomic differences associated with particular flower color traits. In 2018, the transcriptomes of marigold buds was sequenced to develop simple sequence repeat (SSR) markers^15^. In this study, we performed de novo sequencing on the four stages of flower development in both the orange and yellow marigold varieties. The resulting high-quality sequencing data, unigene assembly, and annotations provide new resources for future marigold research and serve as a basis for efforts to improve this important ornamental crop.

‘V-01’ and ‘V-01M’ are isogenic, meaning that most of the DEGs identified, particularly in the pigment biosynthesis pathways, are the consequences or causes of the observed differences in their xanthophyll accumulation. Two enzymes, HYD-B and HYD-E, are crucial for the catalysis of carotene and the production of xanthophylls, including lutein. Knocking out either *HYD-B* or *HYD-E* was previously shown to affect the biosynthesis of lutein in Arabidopsis^16^. Based on our transcriptome analysis, we revealed that the expression levels of *HYD-B* and *HYD-E* gradually increased during the development of the marigold flowers, which was consistent with their pigmentation progression. In both ‘V-01’ and ‘V-01M’, the expression patterns of *HYD-B* and *HYD-E* were similar throughout flower development, with no significant differences between the two genotypes. In contrast, the genes encoding enzymes important for the degradation of the xanthophylls, included two *CCD*s and two *NCED*s, were found to be differently expressed in ‘V-01’ and ‘V-01M’. The dramatically low expression of these four carotenoid degradation genes in the yellow mutant ‘V-01M’ might lead to the high accumulation of all xanthophylls in its petals. Similarly, in strawberry (*Fragaria* × *ananassa*) fruit, previous studies revealed a correlation between the increased expression of *FaCCD1* during ripening and a decrease in the lutein content^9^. In addition, the RNAi-mediated silencing of *CCD4a* in *Chrysanthemum morifolium* increased the lutein content in its petals, changing them from white to yellow^17^.

## Conclusions

Here, we performed an analysis of the transcriptome and metabolites to investigate the molecular and genetic causes of the different flower colors observed in the orange ‘V-01’ marigold line and its isogenic yellow mutant ‘V-01M’. These investigations showed that xanthophylls accumulate in the yellow ‘V-01M’ flowers, and led to the identification of a set of genes involved in carotenoid biosynthesis. We did not identify a significant difference in the expression of the xanthophyll biosynthesis genes *HYD-B* and *HYD-E* between ‘V-01’ and ‘V-01M’; however, the dramatically reduced expression of the *CCD*s and *NCED*s in ‘V-01M’ might limit the degradation of the xanthophylls, resulting in the yellow petal coloration. This work also provides a transcriptome database for the study of marigold, an economically important ornamental plant.

## Methods

### Plant materials

The marigold (*Tagetes erecta* L.) inbred line ‘V-01’ (orange petals) and its natural mutant ‘V-01M’ (yellow petals) (Fig. 1A) were grown in a climate chamber at 22 °C with 70% relative humidity and a 16-h/8-h day/night photoperiod. Flower development was divided into four stages: pre-flowering (I), unopened flower (II), semi-open flower (III), and full flowering (IV) (Fig. 1B). Flowers were harvested from both plants at these four developmental stages, immediately frozen in liquid nitrogen, and stored at – 80 °C until required for the metabolites and transcriptomic analyses. Three replicates were prepared for each sample.

### Pigment extraction

Petals (100 mg fresh weight) were frozen in liquid nitrogen, ground into powder, and extracted with a solution of *n*-hexane:acetone:ethanol (2:1:1, v/v/v). The extract was vortexed for 30 s, then an ultrasound-assisted extraction was carried out for 20 min at room temperature. The extract was centrifuged at 12,000 rpm for 5 min, after which the supernatant was collected and evaporated under a nitrogen gas stream. The extract was then reconstituted in 75% (v/v) methanol and centrifuged, and the supernatant was collected for the liquid chromatography-mass spectrometry (LC–MS) analysis.

### Carotenoid metabolites analysis

The petal extracts were analyzed using an LC–ESI–MS/MS system (high-performance liquid chromatography (HPLC) Shim-pack UFLC SHIMADZU CBM30A system; MS, Applied Biosystems 6500 Triple Quadrupole). The analysis was performed using YMC C30 columns (3 µm, 2 mm × 100 mm) and an acetonitrile:methanol (3:1, v/v) (0.01% BHT):methyl tert-butyl ether (0.01% BHT) solvent. The solvent gradient was as follows: 85:5 (v/v) at 0 min, 75:25 V/V at 2 min, 40:60 (v/v) at 2 min 30 s, 5:95 (v/v) at 3 min, 5:95 (v/v) at 4 min, 85:15 (v/v) at 4 min, and 85:15 (v/v) at 6 min. The flow rate was 0.8 mL/min. The temperature was maintained at 28 °C, and the injection volume was 5 μL.

The effluent was alternatively connected to an ESI-triple quadrupole-linear ion trap (Q TRAP)-MS (API 6500 Q TRAP LC/MS/MS System) equipped with an APCI Turbo Ion-Spray interface operating in a negative ion mode. The equipment was controlled using Analyst 1.6.3 software (AB Sciex). The APCI source operation parameters were as follows: ion source, turbo spray; source temperature 350 °C; curtain gas (CUR) was set at 25.0 psi; and collision gas (CAD) was medium. The DP and CE settings were further optimized for individual MRM transitions. A specific set of MRM transitions were monitored for each period, according to the plant hormones eluted.

### RNA sequencing and de novo assembly

Total RNA was isolated from the petals using an RNAprep Pure kit (Tiangen Biotech Co., Ltd., Beijing, China). The RNA concentration was determined using a NanoDrop 2000 spectrophotometer (Thermo Fisher Scientific, Waltham, MA, USA) and an Agilent 2100 Bioanalyzer (Agilent Technologies, Santa Clara, CA, USA). RNA samples with an RNA integrity number (RIN) greater than 9 were used to construct the sequencing library using a NEB Next Ultra Directional RNA library prep kit, according to the manufacturer’s instructions (New England Biolabs, Ipswich, MA, USA). The library preparations were sequenced on an Illumina Hiseq 2000 platform by ORI-GENE Technology Inc. (Beijing, China). The raw sequencing data were filtered to remove low-quality reads that could affect the data quality and subsequent analysis. To this end, the reads were cleaned using FastQC software to remove the adaptors and poly-N-containing low-quality reads, as well as reads shorter than 40 bp. The transcriptome was assembled using Trinity software with the min_kmer_cov set to 2, and all other parameters set to their defaults. The assembled transcripts and unigenes were used for the subsequent annotation analysis.

### Differential expression analysis

The unigenes were functionally annotated by searching for homologs in a comparison against the public databases Swiss_Prot, Translated EMBL (TrEMBL), Non-redundant Proteins (NR), Protein Families Database (Pfam), Eukaryotic Orthologous Groups Database (KOG), Gene Ontology (GO), and Kyoto Encyclopedia of Genes and Genomes (KEGG)). The number of fragments per kilobase of transcripts per million mapped reads (FPKM) was calculated for the quantification of the gene expression levels. The abundance of the gene transcripts was calculated using Cufflinks. The cuffdiff command was used to filter out the differential genes with a mapping read sum greater than 10 in both samples, |log2 (fold change)| > 1, P-value ≤ 0.05, and Q-value ≤ 0.05. GO and KEGG significant enrichment analyses was performed, and a hypergeometric test (phyper) was used to identify any GO/KEGG terms that were significantly enriched in the differentially expressed genes (DEGs) compared with all of the expressed genes (P-value < 0.05).

## Supplementary information

Supplementary Figure S1.

Supplementary Information.

Supplementary Table S1.

Supplementary Table S2.

## Data Availability

The sequencing raw data of this study was deposited in NCBI database (BioProject PRJNA562616). The plant materials are available from the corresponding author on reasonable request.

## Abbreviations

MEP: 2-C-methyl-d-erythritol-4-phosphate pathway
DEGs: Differentially expressed genes
DXS: 1-Deoxylulose-5-phosphate synthase
DXR: 1-Deoxylulose-5-phosphate reductionomerase
GGPPS: Geranylgeranyl pyrophosphate synthase
PSY: Phytoene synthase
PDS: Phytoene desaturase
Z-ISO: 15-Cis-ζ-carotene isomerase
ZDS: ζ-Carotene desaturase (ZDS)
CRTISO: Carotenoid isomerase
LCY-B: β-Cyclase
HYD-B: β-Hydroxylase
LCY-E: ε-Cyclase
HYD-E: ε-Hydroxylase
CCD: Carotenoid cleavage dioxygenase
NCED: 9-*cis* Epoxy carotenoid cleavage dioxygenase
