# Supplementary figures and images for: Carotenoid metabolite and transcriptome dynamics underlying flower color in marigold (Tagetes erecta L.)
Source: Sci Rep. 2020 Oct 8;10:16835. doi: 10.1038/s41598-020-73859-7 (PMC7544827; doi:10.1038/s41598-020-73859-7)

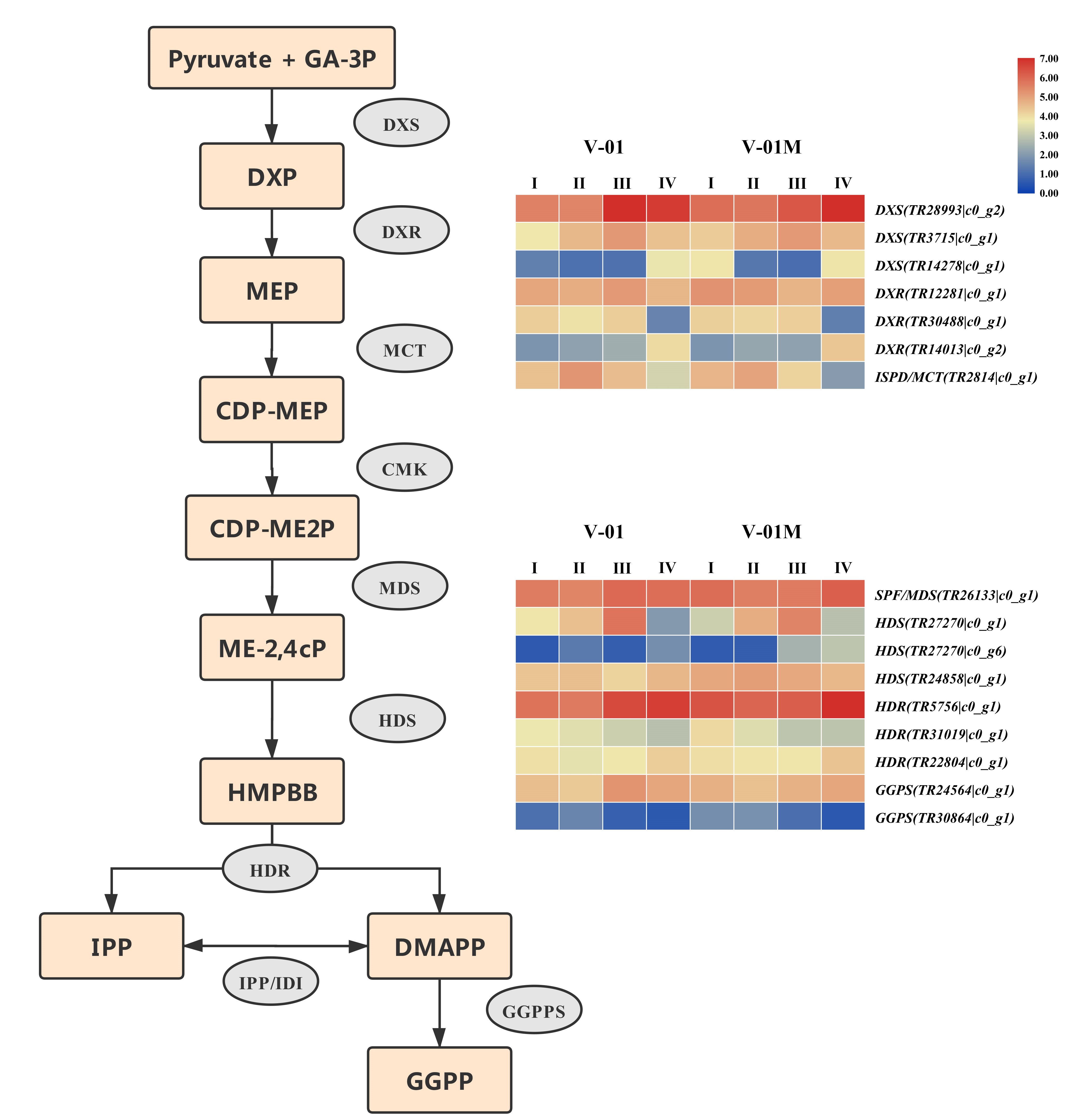

Supplement: Supplementary file 1 — Supplementary Figure S1. [file 41598_2020_73859_MOESM1_ESM.jpg]
