# Supplementary material for: Carotenoid metabolite and transcriptome dynamics underlying flower color in marigold (Tagetes erecta L.)
Source: Sci Rep. 2020 Oct 8;10:16835. doi: 10.1038/s41598-020-73859-7 (PMC7544827; doi:10.1038/s41598-020-73859-7)
Supplement: Supplementary file 2 — Supplementary Information. [file 41598_2020_73859_MOESM2_ESM.pdf]

SUPPLEMENTARY INFO

Carotenoid metabolite and transcriptome dynamics underlying flower color in marigold (*Tagetes erecta* L.)

Running head: Color formation in marigold flowers

Huali Zhang[1#], Shiya Zhang[2#], Hua Zhang[1], Xi Chen[3], Fang Liang[1], Helan Qin[1], Yue Zhang[1], Richen Cong[1], Haibo Xin[1*] and Zhao Zhang[2*]

Authors Affiliations:

[1]Beijing Key Laboratory of Greening Plants Breeding, Beijing Institute of Landscape Architecture, Beijing, China

[2]Beijing Key Laboratory of Development and Quality Control of Ornamental Crops, Department of Ornamental Horticulture, China Agricultural University, Beijing, China

[3]Jiangsu Vocational College of Agriculture and Forestry, Nanjing, China

[#]These authors contributed equally

[*]**Authors for correspondence:**

Haibo Xin, Beijing Institute of Landscape Architecture, Beijing 100102, China. e-mail: zghhzpx@163.com

Zhao Zhang, Department of Ornamental Horticulture, China Agricultural University, Yuanmingyuan Xilu 2, Beijing 100193, China. Tel. 0086-10-62733603, e-mail: zhangzhao@cau.edu.cn

SUPPLEMENTARY DATA

Supplemental Figure S1. Expression patterns of genes encoding enzymes putatively involved in the early 2C-methyl-D-erythritol 4-phosphate (MEP) pathway. Gene expression levels in the four flower developmental stages in both marigold ‘V-01’ and ‘V-01M’ are represented by heat maps. DXS, 1-Deoxy-D-xylulose 5-phosphate synthase; DXR, 1-Deoxy-D-xylylose 5-phosphate reductoisomerase; MCT, 2-C-Methyl-D-erythritol 4-phosphate cytidylyltransferase; CMK, 4-(Cytidine 50 -diphospho)-2-C-methyl-D-erythritol kinase; MDS, 2-C-Methyl-D-erythritol 2,4-cyclodiphosphate synthase; HDS, 4-Hydroxy-3-methylbut-2-enyl diphosphate synthase; HDR, 4-Hydroxy-3-methylbut-2-enyl diphosphate reductase; GGPS, Geranylgeranyl diphosphate synthase.

Supplementary Table S1. Quality analysis of all reads from 24 samples.

Supplementary Table S2. Number of DEGs from all comparisons
